# Cupin: A candidate molecular structure for the Nep1-like protein family

**DOI:** 10.1186/1471-2229-8-50

**Published:** 2008-04-30

**Authors:** Adelmo L Cechin, Marialva Sinigaglia, Ney Lemke, Sérgio Echeverrigaray, Odalys G Cabrera, Gonçalo AG Pereira, José CM Mombach

**Affiliations:** 1 Programa de Pós-Graduação em Computação Aplicada, Unisinos, Av. Unisinos – 950, São Leopoldo, Brasil; 2 Departamento de Física e Biofísica, UNESP, Dist. Rubião Jr. sn, Botucatu, Brasil; 3 Instituto de Biotecnologia, UCS, R. Francisco Getúlio Vargas 1130, Caxias do Sul, Brasil; 4 Departamento de Genética e Evolução, IB/UNICAMP, Campinas, Brasil; 5 Centro de Ciências Rurais, UFPampa/UFSM, São Gabriel, Brasil

## Abstract

**Background:**

NEP1-like proteins (NLPs) are a novel family of microbial elicitors of plant necrosis. Some NLPs induce a hypersensitive-like response in dicot plants though the basis for this response remains unclear. In addition, the spatial structure and the role of these highly conserved proteins are not known.

**Results:**

We predict a *3d*-structure for the *β*-rich section of the NLPs based on alignments, prediction tools and molecular dynamics. We calculated a consensus sequence from 42 NLPs proteins, predicted its secondary structure and obtained a high quality alignment of this structure and conserved residues with the two Cupin superfamily motifs. The conserved sequence GHRHDWE and several common residues, especially some conserved histidines, in NLPs match closely the two cupin motifs. Besides other common residues shared by dicot Auxin-Binding Proteins (ABPs) and NLPs, an additional conserved histidine found in all dicot ABPs was also found in all NLPs at the same position.

**Conclusion:**

We propose that the necrosis inducing protein class belongs to the Cupin superfamily. Based on the *3d*-structure, we are proposing some possible functions for the NLPs.

## Background

More than 10 years ago, a 24-kD necrosis and ethylene inducing protein, named NEP1, capable of triggering plant cell death was purified from culture filtrates of *Fusarium oxysporum*. Since then, several other NEP1-like proteins (NLPs) have been identified in diverse microorganisms; including bacteria, fungi, and oomycetes [1]. In several cases, one species have more than one copy of NLPs and it is believed that several of these copies are pseudogenes [2,3]. NLPs constitute a family of phytotoxic proteins that contains a secretory signal sequence and are able to elicit cell death and defense responses in a large number of dicot plants (reviewed by [4] and [2]). Most species with NLPs are plant pathogens but there are exceptions, since genes encoding NLPs have been detected in fungal and bacterial species that are not known to be pathogenic.

A recently published study identified three copies of NLPs in the basidiomycete *Moniliophthora perniciosa *(MpNEPs). *M. perniciosa*, the causal agent of the witches' broom disease in *Theobroma cacao*, is responsible for major crop losses in the Americas. The authors observed that despite the high sequence similarity, MpNEP1 and MpNEP2 present different structural features, and MpNEP2 activity was resistant to high temperatures. They also demonstrated that these genes are differentially expressed in two different life stages of the fungus [5].

All NLPs contain a conserved domain called necrosis-inducing *Phytophthora *protein 1 (NPP1) [6]. The current lack of knowledge about functional domains, cellular targeting or protein binding motifs in this type of proteins complicates the unveiling of the actual function of NLPs [4]. There is an increasing interest in the determination of their function, role in plant-pathogen interactions and molecular structure [4]. The main conserved motif GHRHDWE shows no significant similarity to any currently known protein sequence and so provides no clues to NLPs function.

The Cupin superfamily was identified by Dunwell in 1998 [7] and is among the most functionally diverse folding described to date, comprising both enzymatic and non-enzymatic members. These include helix-turn-helix transcription factor, AraC type transcription factor, oxalate decarboxylase, auxin-binding protein, globulins, etc. Many proteins on this superfamily have functions and chemical properties related to the NLPs: Auxin-Binding Proteins (ABPs) are hormone receptors and have a great influence on plant physiology; the related oxalate oxidase is involved in pathogen activities and germin-like proteins, apoplastic, glycoproteins are remarkably protease-resistant because of their cupin fold.

According to Dunwell et al. [8,9] the cupin domain comprises two conserved motifs, each corresponding to two *β-strands*, separated by a less conserved region composed of another two *β-strands *with an intervening variable loop. The total size of the inter motif region varies from 11 residues to ca. 50 residues. The characteristic conserved sequence in motif 1 and 2 is g(x)_5_hxh(x)_3,4_e(x)_6_g and g(x)_5_pxg(x)_2_h(x)_3_n, respectively.

## Introduction

Homology searches using the NCBI-Blast produces no useful results in relation to the *3d*-structure because the possible candidates have such a low score that they cannot be considered viable candidates. The result is a long list of necrosis and ethylene-inducing proteins, all of which are *β-sheet*-rich proteins, but none with useful information with associated *3d*-structure. Any attempt to find other similar proteins based on their *1d*-structure (sequence) to NLP protein results in other NLP proteins. In this article, we propose a *3d*-structure for this protein family based on: (1) *1d*-structure and conserved residues, (2) the supposed catalytic center, (3) the predicted signal sequences and target location, (4) cysteine and histidine conserved residues, and (5) the predicted *2d*-structures. Our computational experiments in association with experimental clues point to the Cupin superfamily as the structure of the NLPs.

**Figure 1:**
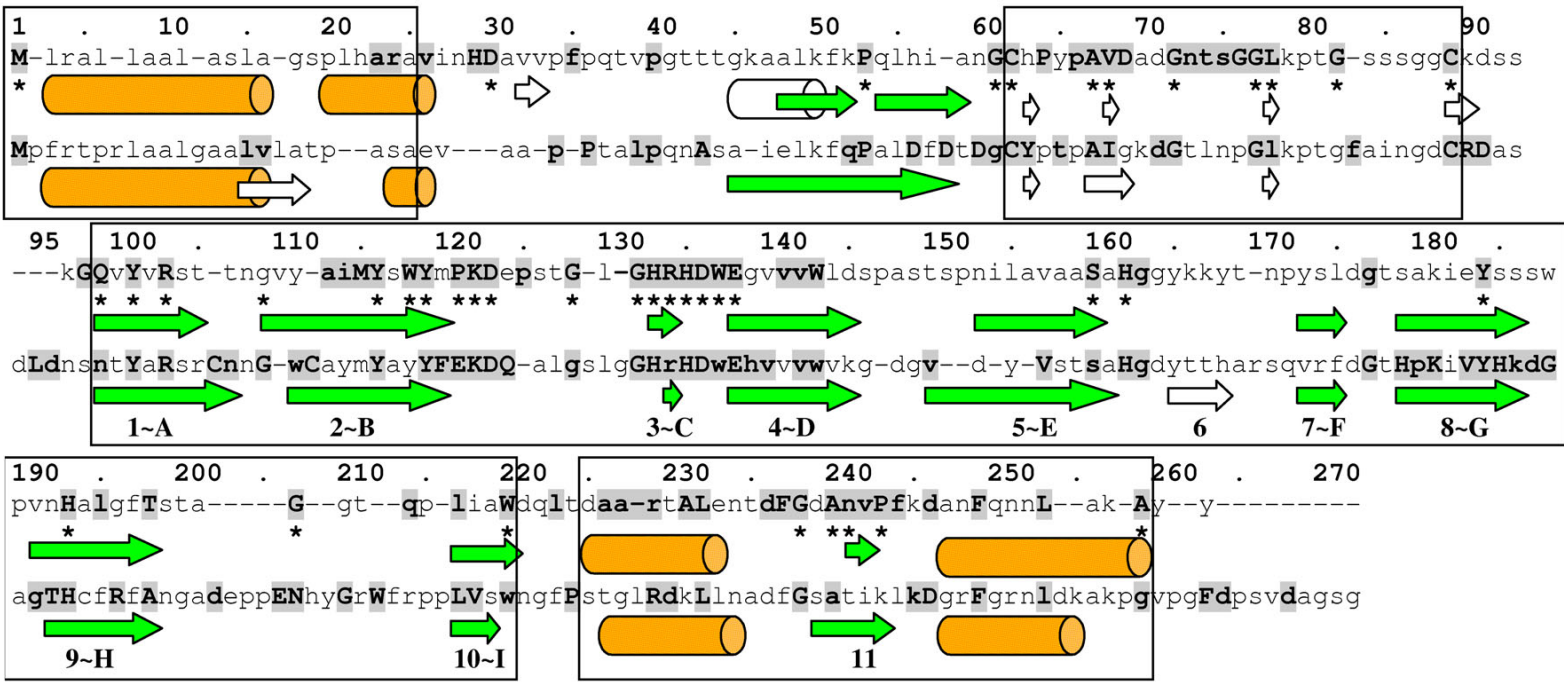
**Consensus sequence and secondary structure prediction of NLPs**. Consensus of all 27 type I NLPs (upper line) and 15 type II NLPs (lower line). Residues present in more than 85% of all sequences are in boldface and capitalized, residues present in more than 70% are in boldface and other residues are in more than 50% of all sequences. Cylinders represent *α-helices *and arrows *β-strands*. The *2d*-structure predictions shown have a level of confidence greater than 33%. White cylinders and arrows represent low confidence level *2d*-structures. Asterisks denote invariant residues (100% conserved) in type I NLPs with experimentally verified necrotic activity.

The article is divided as follows. In section Methods we present the sequences chosen for the analysis and the results of NLPs alignments concerning the conserved residues. Based on the pattern of conserved residues, we looked for candidate structures taking into account also the predicted *2d*-structure. The candidates selected were those with the best agreement in *2d*-structure and conserved residues with NLPs. With these we are proposing a *3d*-structure for the core region (*β-strand*-rich region, positions 90–220 in Figure 1) of the type I NLPs. Based on this proposal; we analyze the most central part of the NLPs and discuss its relation to known proteins.

## Results and Discussion

### Alignment Analysis

Gijzen and Nürnberger classify NLPs into two groups: those containing two cysteines (type I NLPs) and those containing four (type II NLPs). Type I NLPs occur in fungi, oomycetes and bacteria while type II NLPs do not occur in oomycetes [2]. Sequence alignment differentiated the NLPs into these two main groups.

The statistical approach presented here parallels the different levels of phytopathogenicity shown by NLPs. They affect different species at different levels of intensity, being host specific.

The result of the alignment analysis of type I and II NLPs is shown in Figure 1. The first sequence represents all type I NLPs and will be called type I NLP consensus, the second sequence represents all type II NLPs and will be called type II NLP consensus. Because these consensus sequences statistically represent type I and II NLPs, we use them to obtain secondary structure predictions and to perform local alignments with proteins with known *3d*-structure. Finally, we used the type I NLP consensus to build a *3d*-structure. After obtaining type I and II consensus sequences, we submitted them to the PROF program in the PredictProtein site [22] to obtain a secondary (*2d*) structure prediction (see Figure 1).

We observed that the NLP *2d*-structure may be divided into 5 parts or domains: (1) a signal peptide (positions 1–25) with an *α-helix*; (2) a start domain (positions 25–60) with 2–3 predicted *β-strands *and a predicted *α-helix *with low confidence level; (3) a *coil *flanked by two cysteines, C62 and C89, called c62c89-*coil*; (4) a *β-strand*-rich region (positions 90–220), composed by 9–10 *β-strands *and (5) an end domain (positions 220–270) with two predicted *α-helices *separated by a *β-strand*. The predicted central *β-strand*-rich region in NLPs included them in the *all-β *SCOP class of proteins.

In this article, we are proposing that the cupin fold is a suitable template for this region (residues 90–220) of the NLPs. The signal peptide is cut away from the sequence and the other regions play a secondary role in the main structure of the NLPs.

According to the literature, the difference between type I and II NLPs are cysteines C106 and C112, present only in type II NLPs [2]. However, we observed other differences, both in the conservation pattern of residues and in the *2d*-structure. For example, while histidine H29 and aspartate D30 are conserved among type I NLPs, they are underrepresented in type II NLPs. Also, the conserved sequence DxDxDgCY (positions 56–63) and the conserved histidines H179 and H185 in type II NLPs (not present in type I NLPs) is intriguing. Concerning the *2d*-structure, the main difference is *β-strand *6, not predicted in type I NLPs. In order to investigate which residues are essential in NLPs, in the sense that only them (and no other) could play an specific role (function and structure), and which of them may be substituted by other compatible ones (for example, same charge, hydropathicity, *α-helix *or *β-strand *bias, etc.), we count the number of common residues in each position of the alignment and draw them in a succession of histograms. For instance, in type I NLPs the conserved motif GHRHDWE is described by: [g^89%^a^7%^k^4%^] [h^96%^y^4%^] [r^100%^] [h^100%^] [d^92%^f^4%^y^4%^] [w^100%^] [e^100%^], where the letter represent the one letter code and the number is the frequency of the aa at this position. For type II NLPs the sequence of histograms is [g^87%^n^13%^] [h^100%^] [r^74%^k^13%^t^13%^] [h^100%^] [d^100%^] [w^80%^f^13%^l^7%^] [e^100%^]. We can see above that the first glycine may be substituted by alanine in type I NLPs. This means that a small flexible residue in this position fulfills (though glycine is more suitable) the necessary role for the structure and function of the NLPs.

We submitted all sequences in positions 132–138 and 132–139 in all the 42 NLPs to the search service of the Protein Data Bank (PDB) and obtained the following list of candidates ordered by e-value (see Methods): *1vj2 *(e-value = 1.0), *1f51 *(2.2), *1ixm *(2.3), *2ftk *(2.3), *1qtr *(2.6), *1wm1 *(2.6), *1x2b *(2.6), *1x2e *(2.6), *2c0h *(2.7) and *2hi0 *(3.9).

The *1vj2 *structure, a protein with unknown function from *Thermotoga maritima *(a thermophilic Eubacteria with an optimum growth temperature of 80°C) belongs to the RmlC-like cupin SCOP superfamily, and the Mainly Beta CATH class. From the *2d*-structure analysis, NLPs were recognized as *β-sheet*-rich structures [19], possibly belonging to the *all-β *SCOP class of proteins of which the RmlC-like cupin is a superfamily. *1vj2 *presents a compatible number of *β-strands *with those of NLPs, their position relative to conserved residues is the same and finally its sequence rhshpwe is very similar to the pattern GHRHDWE of the NLPs. *1vj2 *has four histidines acting as ligands for a manganese ion, what would explain the importance of this motif in the NLPs. The other candidates, *1f51*, *1ixm *and *2ftk *present the sequence ghsrhdwm in the middle of an *α-helix *and for that they were discarded. Further, all the proteins *1qtr*, *1wm1*, *1x2b*, *1x2e*, *2c0h *and *2hi0 *posses many *α-helices *intermixed by few *β-strands*, and were discarded too.

**Figure 2:**
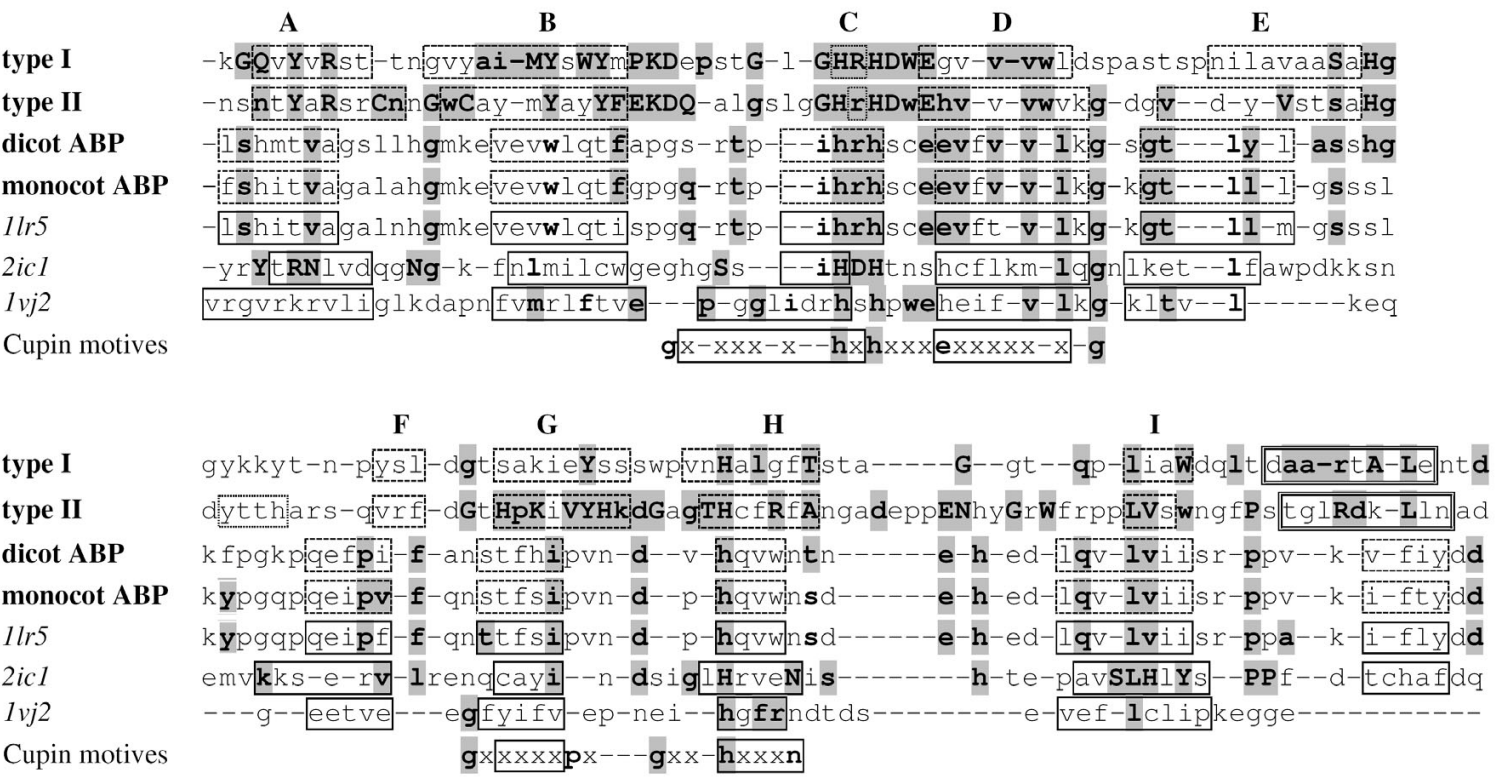
**Sequence alignment of the *β*-barrel domain of NLPs, ABPs and some cupins**. Alignment of the consensus sequence of all 27 type I NLPs (first line), 15 type II NLPs (second line), 32 dicot ABPs (third line), 9 monocot ABPs (fourth line), *1lr5 *(maize ABP), *2ic1 *(cysteine dioxygenase type 1, capitalized residues represent 100% conservation in 10 different organisms), *1vj2 *(hypothetical protein) and the two main cupin motifs (last line). Solid line boxes represent real *β-strands*, dashed line boxes represent those predicted and dotted line boxes are predicted *β-strands *with low confidence level. Compatible residues are shown in boldface and those residues present in both type I/II NLPs and any of the other sequences are grey boxed. The first two lines follow the convention of Figure 1.

We investigated the degree of conservation of the residues in these two motifs in 68 cupins collected in a review by Dunwell [8] and we obtained the following histograms:

$$\begin{array}{c} {\text{g}}^{56\%}{\text{xxxxxh}}^{82\%}{\text{xh}}^{65\%}\text{xxx}[\text{x}-]{\text{e}}^{53\%}{\text{xxxxxxg}}^{97\%}\\ {\text{g}}^{81\%}{\text{xxxxxp}}^{90\%}{\text{xg}}^{68\%}{\text{xxh}}^{75\%}{\text{xxxn}}^{47\%} \end{array}$$

where we can see the typical positioning of the histidines enabling them to act as ion ligands [23]. For the positioning of these motifs in the *2d*-structure or relative to the other *β*-strands, see Figure 2, last line. These two motifs (more exactly, all three histidines) are near each other in the *3d*-structure, enabling the 3 histidines (h^82%^, h^65%^, h^75%^) and the glutamate (e^53%^) to act as metal ligands, what might explain why these residues are highly conserved (see Table 1). Some cupins (called *3*-residue) have three residues between the second and third ligands while others have four (*4*-residue cupins).

**Table 1:** Statistics for NLPs and cupins.

**Position**	**132**	**133 l^st ^ligand**	**134**	**135 2^nd ^ligand**	**136**	**137**	***gap***	**138**	**139 3^rd ^ligand**	**193 4^th ^ligand**
type I NLPs	**g**^89^a^7^k^4^	**h**^96^y^4^	**r**^100^	**h**^100^	**d**^92^f^4^y^4^	**w**^100^	-	**e**^100^	**g**^33^n^30^h^19 ^a^11^y^7^	**h**^92^a^4^p^4^
type II NLPs	**g**^89^n^13^	**h**^100^	**r**^74^k^13^t^13^	**h**^100^	**d**^100^	**w**^80^f^13^l^7^	-	**e**^100^	**h**^80^n^20^	**h**^100^
3-residue cupins	**e**^30^p^24^a^9^l^9^i^9^x^20^	**h**^85^q^6^x^9^	**r**^18^l^18^h^15 ^y^12^q^9^x^27^	**h**^70^d^12^x^18^	**d**^24^t^18^e^12 ^p^9^x^36^	**d**^27^a^18^y^12 ^s^9^x^33^	-	**e**^27^d^24^a^15^x^33^	**e**^39^a^18^v^15^n^6^h^6^x^15^	**h**^76^f^9^m^6^y^3^l^3^s^3^
4-residue cupins	**p**^34^l^31^x^34^	**h**^80^q^14^x^6^	**y**^31^w^17^i^9^k^9^r^9^x^25^	**h**^60^n^14^x^26^	**p**^23^s^17^q^9^x^51^	**h**^17^n^11^r^11^d^6^q^9^x^43^	**a**^29^r^17^s^17^q^11^h^9^x^17^	**d**^23^t^17^s^11 ^a^9^e^9^x^31^	**e**^66^k^9^v^9^l^6^a^3^g^3^q^3^t^3^	**h**^74^f^9^V^9^q^6^m^3^

Statistics for 27 type I NLPs, 15 type II NLPs, 33 *3*-residue cupins and 35 *4*-residue cupins. Values are given as percentage of these numbers. x means any other residue.

Comparing the residue histograms of NLPs and cupins in Table 1, we can see that the sequence hrhdxe is present in most NLPs and in most *3*-residue cupins. Also, 75% of all cupin sequences and 95% of all NLPs have a histidine (fourth ligand) in the second motif and at position 193, respectively. These correspond to the most important residues in the general cupin pattern and, the substitution of any of these residues will reduce the ability of the protein to hold the metal ion, as is the case in some cupins. We concluded that the first motif in the cupins with its xhxhxxx [x-]e pattern corresponds to the GHRHDWE [gh] pattern of the NLPs and the histidine h^75% ^in the second cupin motif corresponds to H193^95% ^in the NLPs. Certainly this correspondence must be compatible with the *2d*-structure, what we will see next.

**Figure 3:**
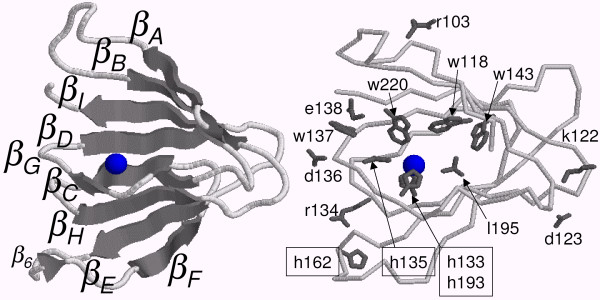
**Three-dimensional structure prediction for the type I NLPs**. Representation of the two *β-sheets *CHEF and ABIDG (left side) and the relative position of some of the conserved residues in the *3d*-structure (right side). The sphere in the middle of the structure represents the putative metal ion.

The embedding of the sequence GHRHDWE in a *β-strand *imposes an alternate orientation (inwards and outwards) of the side chains. Furthermore, the hydrophobicity pattern must be compatible with that fact. Highly hydrophobic residues, such as tryptophan (w), extend their hydrophobic side chains toward the interior the protein, inducing the orientation g-h133-r↑-h135-d↑-w↓-e↑-[gh]-v-v-v-w↓ (a down arrow represents sidechain directed toward the interior of the protein and an up arrow the opposite). Histidines H133 and H135 obey this alternate pattern in the NLPs allowing them to act as ligands for metal ions (see Figure 3).

In cupins, both positions 138 and 139 (see Table 1) typically contain negatively charged residues, such as aspartate (d) or glutamate (e). However, only E139 acts as a ligand for the metal ion. Therefore, although highly conserved in type I and II NLPs, e138 must be discarded as a viable ligand candidate. h139 could act as an ion ligand in type II NLPs, but only 19% of type I NLPs presents a histidine at this position. Among all 68 cupins in [8], only the sequences from *Pyrococcus horikoshii *and *Arabidopsis thaliana *have a histidine at this position, ihqhdwe*h *(GenBank gi 3256432, a hypothetical protein) and ahhhtfg*h *(gi 1169199, DNA-damage-repair/toleration protein), respectively. The previously obtained *T. maritima 1vj2 *with its sequence r*h*s*h*pwe*h *(ligands are italicized) is included in this group, too. It seems that the third histidine confers an increased stability to the binding of the metal ion necessary in the extreme temperature living conditions of *P. horikoshii *and *T. maritima*.

The second most frequent residue at position 139, asparagine (n), is found only in two cupins among the 68 in [8]: *Arachis hypogaea *(gi 1168390a) pkhadad*n *and *Bacillus subtilis *(gi 2636534) ahfdayt*n*. Because of the lack of histidines in positions 133 and 135, these cupins probably do not bind any metal ion.

Asparagine n139 is present in 26% of the NLPs and probably do not participate in the bind of any ion, too. Additionally, the fact that many NLPs (26%) have non-charged residues at position 139 raises the question if a charged residue is necessary at this position. Many cupins (29%) have uncharged residues (v^8^a^7^l^3^cg) at this position showing that these cupins do not need residue 139 at all as an ion ligand. For example, pirin *1j1l *(d*h*p*h*rgfet...*h*a*e*) uses three histidines, (h133, h135, and h193) and a glutamate (e195) as ligands for Fe^2+^, but not e139, though it would be available to perform this function. Moreover, several cupins do not use any 3^*rd *^ligand at this position. Examples are isopenicillin N synthase from *Aspergillus nidulans*, PDB code *1bk0 *(sequence w*h*e*d*vslit...*h *and ion Fe^3+^); clavaminate synthase *1ds1 *(sequence f*h*t*e*mathr...*h *and ion Fe^2+^); hypothetical protein *1jr7 *(sequence l*h*n*d*gtyvee...*h*, and ion Fe^2+^) and anthocyanidin synthase from *Arabidopsis thaliana 1gp4 *(a*h*t*d*vsaltf...*h*, Fe^3+^). Even when present, e139^53% ^is not very conserved in cupins for a residue that should bind to a metal ion. Additionally, site-directed mutagenesis e139 → q139 in the cupin acetylacetone dioxygenase Dke1 results in increased loss of the Fe^2+ ^ion and reduced thermal stability [24], but its functional characteristics remain practically unchanged. We conclude that asparagine n139 does not act as a ligand for the metal ion, resulting in 61% of all NLPs with no ligand at this position. Finally, human cysteine dioxygenase *2ic1 *(see Figure 2) has just 3 histidine ligands for the Fe^2+ ^ion (h133, h135 and h193). The third histidine in this sequence i*h*d*h*tdshc...*h *does not act as a metal ligand and no other residue is necessary to hold the metal ion showing that NLPs could likewise, hold a metal ion at this site.

### The Role of Cysteines

Fellbrich et al. [6] have shown that both cysteines C62 and C89 are conserved and necessary for the NLPs function. Also, the coil between them seems to encode a glycosylation site, which, for secreted proteins means protection against proteolysis, correct folding and thermal stability. Additionally, the highly conserved glycines G76G77 seem to promote a fold exactly in the middle of this coil enabling the cysteines to come together.

The analysis of the bonding pattern among cysteines resulted in a 90% confidence level for C62 and C89 to be forming a disulfide bridge in type I and II NLPs. A search for the pattern GnxsGGL in the PDB rendered the protein *1eh6*, which has a *turn *at s75G76, supporting the hypothesis that both cysteines are disulfide bonded.

In relation to the other two cysteines present only in type II NLPs, the program DISULFIND attributes a probability of just 30% for the bonding of C106 to any other cysteine and 0% for C112. However, from the position of these two cysteines, it is not difficult to infer that they are bonded if *β*_*A *_and *β*_*B *_form a *β-sheet *(see Figure 3). These two cysteines seem to enforce that these *β-strands *should present this conformation. It is also possible that these two cysteines might be bonded to the two conserved histidines H133 and H134 by a zinc ion, such as in the zinc finger of WRKY proteins. WRKY-proteins have a special zinc-finger motif characterized by the pattern cx_4,5_cx_22,23_hxh, and type II NLPs have a similar pattern, cx_4_cx_19_hxh. WRKY-proteins are transcript factors with up to 100 representatives in *A. thaliana *[25]. For instance, the protein AtWRKY6 is associated with both senescence- and defense-related processes [26]. The structure of the WRKY proteins may be shared by type II NLPs. However, it is less probable that they share the same function. [27] suggests that NLP-induced necrosis requires interaction with a target site at the extracytoplasmic side of dicot plant plasma membrane. They show that the ectopic expression of NLP in dicot plants resulted in cell death only when the protein was delivered to the apoplast. However, Bae et al. have shown that NEP1 in the plant was localized at the cell wall and cytosol. This result indicates that NEP1 can penetrate through the plasma membrane but may not be able to penetrate organelles [28]. It has been observed that NLPs are hydrophilic and not likely to cross the plasma membrane. Furthermore, our proposed model structure based on the ABP *1lr5 *has many hydrophilic residues at the surface and the hydrophobic ones are buried supporting the hypothesis that NLPs are not able to cross the plasma membrane. Additionally, the rapid response of parsley protoplasts (approximately 150 seconds) to PpNPP1 (*Phytophthora parasitica *NPP1) is compatible with an interaction just at the plasma membrane level [6].

### NLP 2d and 3d-Structure

The RmlC-like cupin superfamily belongs to the SCOP double-stranded beta-helix fold. Cupins are double-stranded because they are composed of two sequences of antiparallel strands linked with short turns. If the NLPs are cupins, then there should be a correspondence between the *2d*-structures of cupins and those predicted for NLPs. Cupins are formed by 8–10 *β-strands *called [A]BCDE-FGHI [J].

The formation of the *β*-barrel can be understood in the following way: It starts with E folding over F, then D over G, C over H, and eventually B folds over I:

$$\begin{array}{cc} \begin{array}{c} \overset{B}{\Rightarrow }\overset{C}{\Rightarrow }\overset{D}{\Rightarrow }\overset{E}{\Rightarrow }\\ \overset{I}{⇐}\overset{H}{⇐}\overset{G}{⇐}\overset{F}{⇐} \end{array} & ) \end{array}$$

Finally, this double strand turns like an helix building up a *β-*barrel of two *β-sheets*: CHEF and BIDG with their hydrophobic residues aiming at the interior of the barrel. Inside the barrel, in the hydrophobic pocket, we find the metal ion bound to its ligand, next to the top of the barrel (the bottom is closed by the E and F *β*-strands, see Figure 3). Cupins presenting a catalytic activity bind their substrates on the top of the barrel close to the metal ion at the hydrophobic pocket.

**Figure 4:**
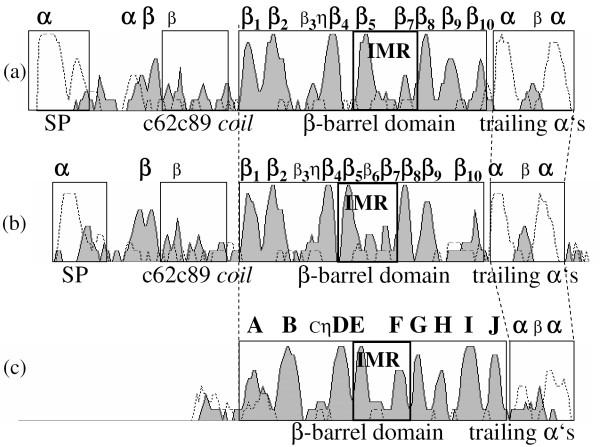
**Confidence level of the PROF prediction**. Representation of the level of confidence of the PROF *2d*-structure prediction for type I NLP consensus sequence (a), type II NLP consensus sequence (b) and *1lr5 *cupin (c). The solid line (shaded gray) represents the confidence level for the *β-strands*, and the dashed line for the *α-helices*. SP = signal peptide. IMR = Inter Motif Region. *η *represents the GHRHDWE motif in type I and II NLP consensus sequences and ihrhscee in *1lr5*, respectively.

The coil between E and F must be flexible enough to allow the folding of EDCB over FGHI. Glycine, as the most flexible residue, represents an excellent candidate to perform this role and we find two of them in the sequence of the putative EF-*coil *in the NLPs: g163g164. Moreover, g163g164 are 27 residues away from the 1^*st *^and 2^*nd *^histidine ligands and 26 residues away from the 4^*th *^histidine ligand in the type I NLPs (H133R134H135-x_27_-g163g164-x_26_-H193). The final result is that all three histidines are very close in the final structure (see Figure 3), exactly as they should be to act as ion ligands. Additionally, an interesting sequence is the necrotic type I NLP BeNEP2 *c*psah g163g164 wd*c *in the EF-*coil*, which is flanked by two cysteines. The DiANNA 1.1 disulfide bond prediction program [29] predicts these two cysteines are bonded with 82% confidence level supporting the above predictions for this coil with the E and F *β-strands *closing the bottom of the barrel. These *β-strands *and the *loop *in between form the so called Inter Motif Region (IMR), which contains 12 to 130 residues and showing no conserved pattern in the cupin. This highly variable region in the cupins and the low confidence level of the *2d*-structure prediction for the NLPs in this region make difficult any *2d*-structure alignment between cupins and NLPs. Figure 4 shows the confidence levels for the *2d*-structure prediction using the PROF program for type I and II NLP consensuses, and for the *1lr5 *cupin, here we can see the correspondence between individual *β-strands *among these proteins. We observe that the *β*-barrel is built up by 7–9 high confidence level *β-strands *and 1–2 low confidence ones with a correspondence between strands in cupins and NLPs. For instance, *β*_1 _in type I NLP consensus corresponds to *β*_*A *_in cupins, *β*_2 _to *β*_*B*_, *β*_3 _to *β*_*C*_, *β*_4 _to *β*_*D*_, *β*_5 _to *β*_*E*_, *β*_7 _to *β*_*F*_, *β*_8 _to *β*_*G*_, *β*_9 _to *β*_*H *_and finally *β*_10 _corresponds to *β*_*I*_. The most conserved pattern in NLPs, the GHRHDWE sequence (*η *in Figure 4), is between the putative C (*β*_3_) and D (*β*_4_) *β-strands*. From the position of this pattern, despite the fact that C is a low confidence strand, its position can be easily determined. This correspondence is confirmed by the alignment of the *β*-barrels of some representative NLP and cupin sequences (see Figure 2). These are the consensus of 27 type I and 15 type II NLPs, 32 dicot ABPs (all cupins), 9 monocot ABPs (all cupins), and three other cupins discussed in this work: *1lr5 *(ABP1), *2ic1 *and *1vj2*.

Two differences between the predictions obtained for type I and II NLP consensuses and the cupin structure are worth mentioning: first, *β*_6 _is present in type II NLPs but not in type I NLPs, and second, NLPs do not have *β*_*J*_. Certainly, the correspondence of *β*_6 _to *β*_*F *_is a tempting assumption in type II NLPs, but this would not be compatible with the extremely good alignment between type I and II NLPs and with the alignment shown in Figure 2. We could argue that the corresponding *β-strand *was just missed by the PROF program in type I NLP consensus and that *β*_8_, and not *β*_9_, should correspond to *β*_*H *_in type I NLPs. Contrary to this idea, we propose that the conserved histidine H193^95% ^acts as metal ion ligand. In cupins, since the 4^*th *^ligand is in *β*_*H*_, we propose H193 signs the position of the H *β-strand *in NLPs. More precisely, the 4^*th *^ligand (histidine) must be at the border of the *β*_*H *_because the 1^*st *^and 2^*nd *^ligands are at the border of *β*_*C *_(in the CD-*coil*) and C and H *β-strands *form an antiparallel *β-sheet*, as can be visualized by the following design (boxes represent *β-strands*).

$$\begin{array}{ccc} \beta_{B} & \beta_{C} & \beta_{D}\\ \ldots \Rightarrow & \text{tglgh}\begin{array}{c} \text{rh} \end{array}\text{dwe} & \Rightarrow \ldots \\ \ldots ⇐ & \begin{array}{c} \text{tfglahnv} \end{array}\text{pg} & ⇐\ldots \\ \beta_{I} & \beta_{H} & \beta_{G} \end{array}$$

Lastly, we could argue that the *3d*-structure of type II NLPs includes 10 *β-strands *and not 9 as in type I NLPs and that *β*_8 _is *β*_*H *_with H179^87% ^(at the border of *β*_8_) being the residue acting as 4^*th *^ligand. Besides the conserved histidines of type I NLPs, type II NLPs have two additional ones: H179^87% ^and H185^100%^, which could act as ligands. First, the good alignment of type I NLPs and type II NLPs points to a common structure, second, most NLPs are type I, third, they include the most aggressive NLPs (necrotic ones), and fourth, type II NLPs do not occur in oomycetes (see Table 2). Therefore, type I NLPs represents the class of the NLPs and type II NLPs should be treated as an important but secondary source of information about the NLP structure.

**Table 2:** Analyzed NLP sequences.

	**Plant pathogens organism**	**GenBank accession number (NLP type)**
Bacteria	*Enwinia carotovora atroseptica*	CAG75986^••II ^(NipEca) [1 0]
Fungi	*Fusarium oxysporum*	AAC97382^•• ^(NEP1) [11] [12]
	*Magnaporthe grisea*	EDK02987^II^, EDJ98732^II^, EDJ96934 and EDJ94825 [13]
	*Verticillium dahliae*	AAS45247^•• ^(His_VdNEP) [14]
	*Botrytis elliptica*	CAJ98683^•• ^(BeNEPl) and CAJ98684 (BeNEP2) [15]
	*Botrytis tulipae*	ABB43261
	*Botrytis fabae*	ABB43270
	*Gibberella zeae*	XP 386193, XP 383570^II^, XP 387963^II ^and XP 391669^II ^[16]
	*Moniliophthora pemiciosa*	ABQ53551^•• ^(Mp NEP1) and ABO32369^•• ^(Mp NEP2) [5]
Oomycetes	*Phytophthora infestans*	AAY43363^•• ^(NPP1), AAY43377° (NPP1.2) and AAY43378° (NPP1.3) [17]
	*Phytophthora megakarya*	AAX12401 and AAX12403 [18]
	*Phytophthora parasitica*	AAK19753^•• ^[6]
	*Phytophthora sojae*	AAM48170^•• ^(PsojNIP), AAM48171 and AAM48172 [19]
	*Pythium aphanidermatum*	AAD53944^•• ^(PaNie234) [20]

	**Other organisms**	**GenBank accession number (NLP type)**

Bacteria	*Bacillus halodurans*_	BAB04114^• ^[19]
	*Bacillus licheniformis*	AAU23136
	*Vibrio pommerensis*	CAC40975^•II ^(causes hemolysis) [21]
	*Streptomyces ambofaciens*	CAJ89765^II^
	*Streptomyces coelicolor*	CAB92890^•II ^[19]
	*Streptomyces griseus*	BAF36639^II^
	*Streptomyces tsusimaensis*	ABA59542^II^
	*Saccharopolyspora erythraea*	YP 001105122^II^
Fungi	*Aspergillus fumigatus*	EAL86241 and EAL86501^II^
	*Aspergillus nidulans*	EAA62936
	*Aspergillus niger*	CAK46514
	*Aspergillus oryzae*	BAE63220
	*Aspergillus terreus*	EAU39525^II^
	*Neurospora crassa*	CAF05864^II^

Analyzed NLP sequences. Type II NLPs (15 sequences) are signed with the II symbol. NLPs signed with a single filled circle^• ^and with double filled circles^•• ^cause a weak and a strong necrosis respectively. An empty circle° signs NLPs reported not to cause necrosis.

### The Inter Motif Region (IMR)

The previous analysis about the *2d*-structure of NLPs and cupins shows that predictions for the region delimited by *β*_6 _and *β*_7 _(putative E and F *β-strands*), which corresponds to the low conserved IMR in cupins, is a difficult task. It contains 22–32 (22–28 in type II NLP consensus) residues with 11 (11 in type II NLP consensus) residues in the *coil *of the IMR in type I NLPs and also has the conserved sequence S^88%^a^50%^H^95%^g^74%^. Therefore, we have chosen among the 68 cupins in [8] those that are similar in size. The most similar cupin to the NLPs' IMR is *A. thaliana *gi|461453, a possible a receptor for the hormone auxin. The *3d*-structure of maize Auxin Binding Protein (ABP) has been already determined (*1lrh *and *1lr5 *in PDB, see Figure 2). It has one *β-barrel *domain, is a dimer in solution, has 21 residues in the IMR, and 11 in *coil*. ABPs are involved in cell expansion and are located in the ER lumen, the plasma membrane, and the cell wall [30]. It is ubiquitous amongst green plants [31].

It would be advantageous for the necrosis proteins to have control of the auxin-response in the host, for example changes in protoplast electrophysiology. Auxin induces H^+ ^secretion into the cell wall causing hyperpolarization of the plasma membrane in *Avena *coleoptile cells [32], electrical response in tobacco protoplasts [33], and K^+ ^currents in *Nicotiana tabacum *guard cells [34].

Auxin stimulates the growth of plant cells by regulating the activity of a H^+^-ATPase in the plasma membrane. Proton secretion by this transport enzyme acidifies the cell walls increasing their extensibility. The internal hydrostatic pressure of the cell then extends the walls. In the interaction between *M. perniciosa *and *T. cacao*, it has been suggested an auxin-inducing phase that causes malformation and an auxin-depleting secondary phase that kills the host [35]. Kilaru et al. reported that the increase in auxin coincided with the phase transition of the fungus. This increase could be the effect of enhanced IAA (auxin) synthesis, suppression of IAA oxidase or secretion of IAA by the pathogen [36]. The possibility that the *3d*-structure of NLPs is similar to that of the cupins and, in particular to ABPs, places the NLPs in control of plant auxin receptors and of the resulting ionic currents. Additionally, Haberlach et al. have shown that the balance of cytokinin and auxin was an important factor in maintaining or eliminating resistance in plant tissues. More specifically, they show that *P. parasitica *resistant *N. tabacum *became susceptible under high cytokinin/auxin levels [37]. It is conceivable that NLPs could compete with the natural ABPs resulting in an apparent increase of the cytokinin/auxin ratio in the plant. Other possibility is that NLPs could be auxin oxidases. Krupasager reported that in the dikaryotic stage, *Marasmius perniciosa *produces no significant amount of cytokinins or auxins but auxin-inactivating enzymes such as IAA-oxidase and laccase [38].

Differences between monocot and dicot ABPs (see Table 3) provide an additional important clue because *Phytophthora *species are primarily pathogens of dicotyledonous plants. Monocots are apparently not affected by NLPs [6]. For example, maize and barley do not show any cell death symptoms after infiltration with PpNPP1. For this reason, we investigated which are the differences between monocot and dicot ABPs [31], and if there is a relationship between these differences with conserved residues in the NLPs. These residues are:

**Table 3:** Differences between monocot and dicot ABPs and NLPs. Differences between monocot and dicot ABPs and NLPs (shown in boldface).

	**2*d*-Structure and glycosylation sites ([nx(st)])**																
	
Protein		C62c89-*coli*				β-barrel											
		
**Type I NLP consensus**	ααβαββ	**C**β	**nts**	**L**	**Ck**	β_*A*_	β_*B *_β_*C*_	ηβ_*D*_β_*E*_	**H**	β_*F*_	β_*G*_	**v**	β_*H*_	**T**	β_*I*_	αβ	α
PpAAK19753	αββαββ	cβ	**nts**	**l**	**ck**	β_*A*_	β_*B *_β_*C*_	ηβ_*D*_β_*E*_	**h**	β_6_β_*F*_	β_*G*_	**v**	β_*H*_	**t**	β_*I*_	αβ	α
MpNEPl	αααβ	cβ	**nws**	**l**	**ck**	β_*A*_	β_*B*_β_*C*_	ηβ_*D*_β_*E*_	**h**	β_*F*_	β_*G*_	**v**	β_*H*_	**t**	β_*I*_	α	α
**Type II NLP consensus**	αβαβ	**C**β	**tln**	l	**Cr**	β_*A*_**C**	**Cβ**_*B*_β_*C*_	ηβ_*D*β*E*_	**H**	β_6_β_*F*_	β_*G*_	**g**	β_*H*_	**A**	β_*I*_	α	α
**Dicot ABP**		cβ_A"_	**nis **α	**l**	β_*A*_,**r**	β_*A*_α	β_*B*_β_*C*_	ηβ_*D*β*E*_	**h**	β_*F *_**nst**	β_*G*_	**v**	β_*H*_	**t**	β_*I*β*J*_	αβ	cα
**Monocot ABP**		cβ_*A*"_	**dis **α	**m**	β_*A*_,**i**	β_*A*_α	β_*B*_β_*C*_	ηβ_*D*_β_*E*_	**s**	β_*F *_**nst**	β_*G*_	**p**	β_*H*_	**s**	β_*I*β*J*_	αβ	cα

The dicot glycosylation site nis sequence is not present in the monocot sequences (dis). nts, nst and nws are possible NLP glycosylation sites.

$$\begin{array}{l} \text{monocot ABPs}\\ \text{dicot ABPs}\\ \text{type I NLP}\\ \text{type II NLP} \end{array}\begin{array}{cccccc} \text{d} & \text{m} & \text{i} & \text{s} & \text{p} & \text{s}\\ \text{n} & \text{l} & \text{r} & \text{h} & \text{v} & \text{t}\\ \text{n}73 & \text{L}78 & \text{k}90 & \text{H}162 & \text{v}191 & \text{t}200\\ \text{t}73 & \text{l}78 & \text{R}90 & \text{H}162 & \text{g}191 & \text{g}200 \end{array}$$

We observe a high degree of correspondence between dicot ABP residues and NLP high conserved residues (n73, L78, H162, v191 and t200 in type I NLPs and l78, R90 and H162 in type II NLPs). Certainly, the histidine residue H162 in the EF-*coil *is the most striking difference between monocot and dicot ABPs shared by NLPs. The alignment between 32 dicot ABPs and type I NLPs in the EF-*coil *results in some common residues (see the sequences below) or residues with similar physicochemical characteristics (see Figure 2 for the whole alignment):

$$\begin{array}{cccccc} \text{type I NLPs} & \cdots & \beta_{E} & \text{aSaHggykkyt} & \beta_{F} & \cdots \\ \text{dicot ABPs} & \cdots & \beta_{E} & \text{asshgkfpgkp} & \beta_{F} & \cdots \end{array}$$

For a more detailed analysis, Table 4 shows for the residues of 9 ABP sequences of monocots and 32 ABP sequences of dicots in the EF-*coil*.

**Table 4:** Residue histograms for NLPs and ABPs.

**Position**	**158**	**159**	**160**	**161**	**162**	**163**	**164**	**165**
**Type I NLPs**	**a**^41^s^41^c^18^	**a**^48^p^30^y^7^l^4^m^4^t^4^v^4^	**s**^96^n^4^	**a**^52^g^26^q^11^-^7^y^4^	**h**^92^a^4^y^4^	**g**^70^s^26^n^4^	**g**^52^k^15^d^11 ^s^11^n^4^r^4^t^4^	**y**^52^w^26^f^7 ^h^7^v^7^
**Type II NLPs**	**s**^60^a^33^g^7^	**t**^33^a^27^p^13^w^13^y^13^	**s**^80^t^20^	**a**^47^c^13^n^13^q^13^e^7^k^7^	**h**^100^	**g**^80^k^7^r^7^s^7^	**d**^33^g^20^k^20^s^13^t^13^	**y**^53^f^20^l^13^v^13^
**Dicot ABPs**	**a**^41^l^36^s^16 ^t^6^	**a**^38^s^31^p^31^	**e**^38^s^31^n^25^	**s**^53^t^47^	**h**^100^	**g**^44^a^16^s^16 ^l^13^e^13^	**k**^53^s^25^e^9 ^t^6^n^13^	**f**^44^y^25^t^19^s^9^h^3^
**Monocot ABPs**	**l**^89^m^11^	**g**^100^	**s**^100^	**s**^78^t^22^	**s**^100^	**l**^56^m^44^	**k**^89^p^11^	**y**^100^

Residue histograms for the 27 type I and 15 type II NLPs, 32 dicot ABP sequences and 9 monocot ABP sequences. Each column represents possible residues at that position. Values are given as percentage of these numbers. x means any other residue.

All 32 investigated dicot ABPs and 95% of all NLPs have a conserved histidine H162 at the EF-*coil*. The two NLP sequences which do not have it exactly at this position, but 4 positions downstream (PiNPP1.2 and PiNPP1.3), are inactive forms; probably originated by gene duplication of the most aggressive PiNPP1 from *Phytophthora infestans*. These two are expressed both in the biotrophic as in the necrotrophic phases, while PiNPP1 is expressed only in the latter phase [17].

Fellbrich [6] has shown that the last 8 residues in PiNPP1 may be deleted without loss of activity but not the last 20 residues (... ntdFGd ◇ AnvPmkdgnFlt ◇ kvgnayya). The sequence AnvPmkdgnFlt coincides 100% with the conserved residues in necrotic type I NLPs GxAnxP. It is interesting to observe that the C-terminal seems to be important for NLPs as well as for ABPs. The synthetic peptide from the C-terminal of *Zea mays *ABP wdedcfeaak, the 15-residue *N. tabacum *Nt-abp1 C-terminal peptide (ywdeecyqttswkdel) and the Nt-abp1 itself have been shown to induce hyperpolarization [39]. However, contrary to the effect of auxins, NLPs cause depolarization, alkalization of the surrounding media and K^+^efflux [40].

The histograms of 32 dicot ABPs in the C-terminal resulted in yWDEqCyqtxxKDEL. Although conserved, mutagenesis experiments have shown that the sequence kdel is not important for the activity of ABPs and may be deleted and it is related to the two negative residues DE. Since NLPs do not have such a sequence, it is conceivable that NLPs compete with ABPs causing the previous discussed effects.

Besides ABPs, another candidate similar in terms of IMR size is *Bacillus subtilis *gi|2635598, a hypothetical protein with no determined structure similar to the human cysteine dioxygenase *2ic1*. It is a monomer with an IMR size of **23 **residues and a coil of **11 **residues. [41] has aligned 10 cysteine dioxygenases of different organisms and the 100% conserved and functionally important residues are capitalized in Figure 2 for the sake of comparison with those in NLPs. We observe that some of these residues are among the most conserved in the necrotic type I NLPs: For instance, Y101 and R103 in *β*_*A*_, H133, H135 and H193 (as ion ligands) and D136.

### Glycosylation

Although an immunoassay study used for the detection of sugars in glycoconjugates did not reveal a carbohydrate moiety in PpNPP1 [6], glycosylation sites nxs are present in 64% of all necrotic type I NLPs (x = t^6^v) in the c62c89-*coil*. The glycosylation occurs at asparagine (n) residues in the so called nx(st) sequon and the efficiency of this process depends on the residue x.

Glycosylation is important in most cell-surface and secreted proteins and is often critical for the interaction with other subunits at the cell surface (recognition), protection against proteolytic attack, protein solubility and thermostability. For instance, *P. infestans *has evolved an arsenal of protease inhibitors to overcome the action of plant proteases [42].

The probability of glycosylation (see section Methods) of the sequon ntsg varies between 44 and 62%. For instance, PiNPP1.1 is 44%, PsojNIP 50%, PpNPP1 54% and BeNEP1 62%. Furthermore, MpNEP1 and MpNEP2 are predicted not to be glycosylated because of the bulky tryptophan (w) in their sequon (nws). His VdNEP and BeNEP2 have no sequon.

Additionally, an important difference between monocot and dicot ABPs [31] pointing out that ABPs and NLPs share the same *3d*-structure is that dicot ABPs have a glycosylation site nis next to the beginning of the protein, that is not present in monocot ABPs (dis). This site is also present in several type I NLPs (see Table 3).

## Conclusion

The *3d*-structure of the NLPs remains to be determined experimentally. However, in this paper we presented several evidences indicating that they belong to the Cupin superfamily. Using a cupin template and the type I NLP consensus we were able to calculate a prediction for the *3d*-structure of the *β-strand *rich portion (positions 90–220 in Figure 1) which presented stability for 3nS under molecular dynamics. This structure presents the classical signature of a cupin protein. Furthermore, the prediction of the structure of the upstream coil bordered by two cysteines remains to be addressed. Cysteines in the upstream coil of type I NLPs are disulfide bonded, simplifying the problem by removing this sequence from the analysis. However, the right positioning of this free coil becomes a new problem, which is more complex than the first one. Is it free to move around or does it make part of the *β-sheets*? Its glycosylation site points to some role in anchoring the protein on the cell surface.

Several *2d*-structure predictions software agree with a central *β-strand *rich portion anked by *α-helices *making highly probable that the central part of the protein belongs to the all-*β *SCOP Class.

The conserved pattern of cysteines points to two different NLP types: type I NLPs containing two cysteines upstream of the *β-barrel *and type II NLPs, containing two additional cysteines in the left border of the *β-barrel*. Predictions show that the first two are bonded together, but not the other two. The similarity between the pattern of cysteines in the WRKY proteins and the type II NLPs is so high, that would not be surprising if the cysteines participate in metal biding in a zinc-finger conformation. Also, WRKY proteins participate in the pathogen detection system of the plant, an interesting "coincidence" for a protein involved in phytopathogenic activities. Unfortunately, we have only part of the WRKY protein structure, the zinc-finger part (C-terminal).

Figure 1 shows clearly the biological role played by the residues of the GHRHDWE motif holding a putatitive ion. For this purpose, a necessary 3^*rd *^histidine is found downstream in the structure (H193) in the exact position according to the cupin structure and is also present in 95% of the NLPs analyzed. Only two NLPs do not have it, and they do not present necrotic activity. The relative position and number of histidines in the structure point to a metal ion containing protein. In addition, the exact metal ion remains to be determined but our study points to manganese or zinc.

The Cupin superfamily is very large with diverse functions. Many of these functions depend on a glutamate residue, which does not seem to be present in NLPs. When this residue is present, cupins are involved in enzymatic activities such as oxalate oxidase, decarboxylase, dioxygenases, etc. It remains to be determined experimentally if NLPs have some catalytic activity involving oxalate. In any case, the [Ca^2+^]_*cyt *_levels, ROS (H_2_O_2_) and oxalate are all intermixed in pathogen defense and sensing. Also, lignin processing is highly dependent on oxidases and peroxidases (cupins). It has been shown that germins function as oxalate oxidases (conversion of oxalate to CO_2 _and H_2_O_2_) and superoxide dismutase ($2{\text{O}}_{2}^{-}$ + 2H^+ ^→ H_2_O_2_+O_2_) [43]. Any interference in such activities would be advantageous for the fungus because of the correlation between H_2_O_2 _and [Ca^2+^]_*cyt*_. Even with no catalytic activity, the fold is resistant to oxidation, a characteristic necessary for oxidases, decarboxylases and peroxidases.

A related class of cupins is the auxin binding proteins, which do not show catalytic activity but work as signal transducers in plant cells. They have many common structural features and conserved residues in relation to the NLPs. In this respect, the most remarkable feature is the conservation of histidine H162, present in 95% of the NLPs and in almost all dicot ABPs and is related to the fact that NLPs attack only dicot plants. Also, the way ABPs transmit the information to the cell is intimately related to ion channels. Correspondingly, the first plant reaction to the NLPs is an increase of ionic currents causing elevation of [Ca^2+^]_*cyt*_. The elevation of H_2_O_2 _(and other ROS species) is upstream and downstream of the [Ca^2+^]_*cyt *_elevation, and both [Ca^2+^]_*cyt *_elevation and H_2_O_2 _are known by their roles in senescence and necrotic activities. Besides all these molecular and functional clues, for example, the fungus *M. perniciosa *causes the formation of witches' broom on *T. cacao *[5]. This reaction is typical of diverse plants in reaction to biotic stresses in its early phase and is related to the cytokinin/auxin balance.

There are several cupin candidates with a compatible IMR size, but only dicot ABPs seem to present some compatibility in relation to size and presence of the conserved histidine in the IMR of the NLPs and predicted glycosylation sites. Certainly, any auxin-like activity over the plant would be advantageous for the fungus (as discussed above). Last of all, as discussed by Fellbrich [6], NLPs are dependent on both, C and N-terminals of the protein for its activity, a feature shared by ABPs.

## Methods

In order to obtain a non-redundant and representative set of NLPs, all sequences analyzed have a pairwise distance greater than 10%. The organisms and sequences include 42 NLPs (see Table 2).

Type I and II NLPs were aligned using ClustalW [44] with default parameters and *gapopen *= 0 (see alignment results in [45] and [46]).

These sequences are obtained computing the most frequent residues in the type I and II NLPs. If, in the alignment of the sequences, a gap is introduced in more than 50% of them, then the respective position is removed from the sequence. Therefore, type I and II NLP consensuses are the consensus of ≥ 50% of the sequences. Furthermore, residues present in more than 85% of all sequences are in boldface and capitalized (see Figure 1). For example, the sequence GHRHDWE occurs in ≥ 85% of all type I NLPs, indicating its important role for the protein function [4]. Residues present in more than 70% of all sequences are in boldface (see Figure 1). For example, the same sequence GHrHDwE shows lower conservation in type II NLPs, as residues r and w occur in < 85% of type II NLP sequences.

We have performed an alignment (ClustalW with *gapopen *= 0) of type I NLPs for which we have experimental evidence of necrotic activity (denoted with one ^• ^and two filled circles ^••^) as shown in Table 2 and we denote the residues conserved in all necrotic sequences with an asterisk in Figure 1 (see alignment results in [47]).

The secondary structure predictions were performed using the PROF program in the PredictProtein site [22].

### Searches in the PDB

The 10 best candidates were obtained by submitting all sequences in positions 132–138 and 132–139 in the 42 NLPs to the search service of the Protein Data Bank (PDB) site [48,49] (Search Tool = *Fasta*). Each sequence generates a list of candidate proteins, which can be ordered by the e-value. The search for local sequences in the PDB was performed using Sequence features = *motif*.

### Disulfide bonds and glycosylation analysis

The analysis of the bonding pattern among cysteines were performed with the DISULFIND program [50]. We also used the DiANNA 1.1 disulfide bond prediction program [29] for the analysis of the BeNEP2. In order to determine the probability of glycosylation of the sequons in the c62c89-*coil*, we submitted all type I NLP sequences to the NetNGlyc 1.0 Server [51].

### Molecular 3d-structure

We used ClustalW with default parameters and *gapopen *= 0.0 to align the sequences shown in the Figure 2. Note that for the alignment, we used only the *β-strand*-rich region (*β*-barrel domain), positions 90–220. The *3d*-structures were constructed with the molecular modelling program *spdbv *[52] using as template the monomer of the *1lr5 *protein. First, we used Rasmol to cut the *β*-rich region of the *1lr5 *protein. Then, we mutated the residues in the *1lr5 *sequence to those residues in the type I NLP consensus, introduced the necessary residues where the alignment resulted in gaps for the *1lr5 *sequence and deleted the residues in the cases the alignment produced gaps in the type I NLP consensus. After each step, we executed a minimization to fit the new residues to the old structure. We took the care to introduce and delete residues only in the coil regions of the protein. The resulting structure was minimized with GROMACS with a steepest descent algorithm until the energy of 0.01 kJ/mol was reached. Then, we performed a simulated annealing from 0 to 30 K during 100 pS in vacuum. The force field used was opls-aa/l. Finally, a simulated annealing of 100 pS from 0 to 10 K and 3 nS from 10 K to 273 K with molecular dynamics in explicit solvent (spc water model) was performed.

## Authors' contributions

GAGP and OGC provided the protein sequences and the main motivation for the work. ALC, MS and JCMM have conceived the study relating the NLP structure and key residues to those of the cupin superfamily. ALC and MS performed the alignments and the analysis of the conserved sequences. ALC performed the *2d*-structure predictions and alignments. ALC and NL calculated the proposed *3d*-structure. SE and MS participated in the proposal of the NLP function. NL, JCMM, and MS made the major revisions of the work. All authors contributed to write the manuscript.

## References

[B1] Bailey BA (1995). Purification of a protein from culture filtrates of *Fusarium oxysporum *that induces ethylene and necrosis in leaves of *Erythroxylum coca*. Phytopathology.

[B2] Gijzen M, Nürnberger T (2006). Nep1-like proteins from plants pathogens: Recruitment and diversification of the NPP1 Domain Across Taxa. Phytochemistry.

[B3] Tyler BM, Tripathy S, Zhang X, Dehal P, Jiang RH, Aerts A, Arredondo FD, Baxter L, Bensasson D, Beynon JL, Chapman J, Damasceno CMB, Dorrance AE, Dou D, Dickerman AW, Dubchak IL, Garbelotto M, Gijzen M, Gordon SC, Govers F, Grunwald NJ, Huang W, Ivors KL, Jones RW, Kamoun S, Krampis K, Lamour KH, Lee MK, McDonald WH, Medina M, Meijer HJG, Nordberg EK, Maclean DJ, Ospina-Giraldo MD, Morris PF, Phuntumart V, Putnam NH, Rash S, Rose JKC, Sakihama Y, Salamov AA, Savidor A, Scheuring CF, Smith BM, Sobral BWS, Terry A, Torto-Alalibo TA, Win J, Xu Z, Zhang H, Grigoriev IV, Rokhsar DS, Boore JL (2006). Phytophthora genome sequences uncover evolutionary origins and mechanisms of pathogenesis. Science.

[B4] Pemberton CL, Salmond GPC (2004). The Nep1-like proteins – a growing family of microbial elicitors of plant necrosis. Mol Plant Pathol.

[B5] Garcia O, Macedo J, Tibúrcio R, Zaparoli G, Rincones J, Bittencourt L, Ceita G, Micheli F, Gesteira A, Mariano A, Schiavinato M, Medrano F, Meinhardt L, Pereira G, Cascardo J (2007). Characterization of necrosis and ethylene-inducing proteins (NEP) in the basidiomycete *Moniliophthora perniciosa*, the causal agent of witches' broom in *Theobroma cacao*. Mycol Res.

[B6] Fellbrich G, Romanski A, Varet A, Blume B, Brunner F, Engelhardt S, Felix G, Kemmerling B, Krzymowska M, Nürnberger T (2002). NPP1, a Phytophthora-associated trigger of plant defense in parsley and Arabidopsis. Plant J.

[B7] Dunwell JM (1998). Cupins: a new superfamily of functionally-diverse proteins that include germins and plant seed storage proteins. Biotechnol Genet Eng Rev.

[B8] Dunwell JM, Khuri S, Gane PJ (2000). Microbial relatives of the seed storage proteins of higher plants: conservation of structure, and diversification of function during evolution of the cupin superfamily. Microbiol Mol Biol Rev.

[B9] Dunwell JM, Culham A, Carter CE, Sosa-Aguirre CR, Goodenough PW (2001). Evolution of functional diversity in the cupin superfamily. Trends Biochem Sci.

[B10] Pemberton CL, Whitehead NA, Sebaihia M, Bell KS, Hyman LJ, Harris SJ, Matlin AJ, Robson ND, Birch PRJ, Carr JP, Toth IK, Salmond GPC (2005). Novel quorum-sensing-controlled genes in *Erwinia carotovora *subsp. *carotovora*: identification of a fungal elicitor homologue in a soft-rotting bacterium. Mol Plant Microbe Interact.

[B11] Jennings JC, Apel-Birkhold PC, Bailey BA, Anderson JD (2000). Induction of ethylene biosynthesis and necrosis in weed leaves by a *Fusarium oxysporum *protein. Weed Sci.

[B12] Bailey BA, Apel-Birkhold PC, Luster DG (2002). Expression of NEP1 by *Fusarium oxysporum *f. sp. *erythroxyli *after gene replacement and overexpression using polyethylene glycol-mediated transformation. Phytopathology.

[B13] Dean RA, Talbot NJ, Ebbole DJ, Farman ML, Mitchell TK, Orbach MJ, Thon M, Kulkarni R, Xu JR, Pan H, Read ND, Lee YH, Carbone I, Brown D, Oh YY, Donofrio N, Jeong JS, Soanes DM, Djonovic S, Kolomiets E, Rehmeyer C, Li W, Harding M, Kim S, Lebrun MH, Bohnert H, Coughlan S, Butler J, Calvo S, Ma LJ, Nicol R, Purcell S, Nusbaum C, Galagan JE, Birren BW (2005). The genome sequence of the rice blast fungus *Magnaporthe grisea *. Nature.

[B14] Wang JY, Cai Y, Gou JY, Mao YB, Xu YH, Jiang WH, Chen XY (2004). VdNEP, an Elicitor from *Verticillium dahliae*, induces cotton plant wilting. Appl Environ Microbiol.

[B15] Staats M, van Baarlen P, Schouten A, van Kan JAL, Bakker FT (2007). Positive selection in phytotoxic protein-encoding genes of Botrytis species. Fungal Genet Biol.

[B16] Han Y, Kim M, Lee S, Yun S, Lee Y (2007). A novel F-box protein involved in sexual development and pathogenesis in *Gibberella zeae*. Mol Microbiol.

[B17] Kanneganti TD, Huitema E, Cakir C, Kamoun S (2006). Synergistic interactions of the plant cell death pathways induced by *Phytophthora infestans *Nep1-like protein PiNPP1.1 and INF1 elicitin. Mol Plant Microbe Interact.

[B18] Bae H, Bowers JH, Tooley PW, Bailey BA (2005). NEP1 orthologs encoding necrosis and ethylene inducing proteins exist as a multigene family in *Phytophthora megakarya*, causal agent of black pod disease on cacao. Mycol Res.

[B19] Qutob D, Kamoun S, Gijzen M (2002). Expression of a *Phytophthora sojae *necrosis-inducing protein occurs during transition from biotrophy to necrotrophy. Plant J.

[B20] Veit S, Worle JM, Nurnberger T, Koch W, Seitz HU (2001). A novel protein elicitor (PaNie) from *Pythium aphanidermatum *induces multiple defense responses in carrot, arabidopsis, and tobacco. Plant Physiol.

[B21] Jores J, Appel B, Lewin A (2003). Cloning and molecular characterization of a unique hemolysin gene of *Vibrio pommerensis *sp. nov.: development of a DNA probe for the detection of the hemolysin gene and its use in identification of related *Vibrio *spp. from the Baltic Sea. FEMS Microbiol Lett.

[B22] Rost B, Yachdav G, Liu J (2004). The PredictProtein Server. Nucleic Acids Res.

[B23] Dunwell JM, Purvis A, Khuri S (2004). Cupins: the most functionally diverse protein superfamily?. Phytochemistry.

[B24] Straganza GD, Eggera S, Aquinoc G, D'Auriac S, Nidetzkya B (2006). Exploring the cupin-type metal-coordinating signature of acetylacetone dioxygenase Dke1 with site-directed mutagenesis: Catalytic reaction profile and Fe2+ binding stability of Glu-69 Gln mutant. Journal of Molecular Catalysis B, Enzymatic.

[B25] Eulgem T, Rushton PJ, Robatzek S, Somssich IE (2000). The WRKY superfamily of plant transcription factors. Trends Plant Sci.

[B26] Robatzek S, Somssich IE (2001). A new member of the Arabidopsis WRKY transcription factor family, AtWRKY6, is associated with both senescence- and defence-related processes. Plant J.

[B27] Qutob D, Kemmerling B, Brunner F, Kufner I, Engelhardt S, Gust AA, Luberacki B, Seitz HU, Stahl D, Rauhut T, Glawischnig E, Schween G, Lacombe B, Watanabe N, Lam E, Schlichting R, Scheel D, Nau K, Dodt G, Hubert D, Gijzen M, Nurnberger T (2006). Phytotoxicity and Innate Immune Responses Induced by Nep1-Like Proteins. Plant Cell.

[B28] Bae H, Kim MS, Sicher RC, Bae HJ, Bailey BA (2006). Necrosis- and ethylene-inducing peptide from *Fusarium oxysporum *induces a complex cascade of transcripts associated with signal transduction and cell death in Arabidopsis. Plant Physiol.

[B29] Ferre F, Clote P (2005). DiANNA: a web server for disulfide connectivity prediction. Nucleic Acids Res.

[B30] Jones AM, Herman EM (1993). KDEL-containing auxin-binding protein is secreted to the plasma membrane and cell wall. Plant Physiol.

[B31] Woo E, Marshall J, Bauly J, Chen J, Venis M, Napier RM, Pickersgill RW (2002). Crystal structure of auxin-binding protein 1 in complex with auxin. EMBO J.

[B32] Cleland RE, Prins HBA, Harper JR, Higinbotham N (1977). Rapid hormone-induced hyperpolarization of the oat coleoptile transmembrane potential. Plant Physiol.

[B33] Warwicker J (2001). Modelling of auxin-binding protein 1 suggests that its C-terminus and auxin could compete for a binding site that incorporates a metal ion and tryptophan residue 44. Planta.

[B34] Bauly JM, Sealy IM, Macdonald H, Brearley J, Dröge S, Hillmer S, Robinson DG, Venis MA, Blatt MR, Lazarus CM, Napier RM (2000). Overexpression of auxin-binding protein enhances the sensitivity of guard cells to auxin. Plant Physiol.

[B35] Evans HC (1980). Pleomorphism in *Crinipellis perniciosa*, causal agent of witches' broom disease of cocoa. Trans Br Mycol Soc.

[B36] Kilaru A, Bailey BA, Hasenstein KH (2007). *Moniliophthora perniciosa *produces hormones and alters endogenous auxin and salicylic acid in infected cocoa leaves. FEMS Microbiol Lett.

[B37] Haberlach GT, Budde AD, Sequeira L, Helgeson JP (1978). Modification of disease resistance of tobacco callus tissues by cytokinins. Plant Physiol.

[B38] Krupasager V, Sequeira L (1969). Auxin destruction by *Marasmius perniciosus*. Am J Bot.

[B39] David K, Carnero-Diaz E, Leblanc N, Monestiez M, Grosclaude J, Perrot-Rechenmann C (2001). Conformational dynamics underlie the activity of the auxin-binding protein, Nt-abp1. J Biol Chem.

[B40] Jennings JC, Apel-Birkhold PC, Mock NM, C J, Baker JDA, Bailey BA (2001). Induction of defense responses in tobacco by the protein Nep1 from *Fusarium oxysporum*. Plant Sci.

[B41] Simmons CR, Liu Q, Huang Q, Hao Q, Begley TP, Karplus PA, Stipanuk MH (2006). Crystal structure of mammalian cysteine dioxygenase: A novel mononuclear iron center for cysteine thiol oxidation. J Biol Chem.

[B42] Tian M, Win J, Hoorn R, Knaap E, Kamoun S (2007). A *Phytophthora infestans *cystatin-like protein targets a novel tomato papain-like apoplastic protease. Plant Physiol.

[B43] Woo EJ, Dunwell JM, Goodenough PW, Marvier AC, Pickersgill RW (2000). Germin is a manganese containing homohexamer with oxalate oxidase and superoxide dismutase activities. Nat Struct Biol.

[B44] Thompson JD, Higgins DG, Gibson TJ (1994). CLUSTALW: improving the sensitivity of progressive multiple sequence alignment through sequence weighting, positions-specific gap penalties and weight matrix choice. Nucl Acids Res.

[B45] Type I NLPs alignment. http://adelmo.cechin.googlepages.com/typeIg0.aln.

[B46] Type II NLPs alignment. http://adelmo.cechin.googlepages.com/typeIIg0.aln.

[B47] Necrotic sequences alignment. http://adelmo.cechin.googlepages.com/tInecg0.aln.

[B48] RCSB Protein Data Bank. http://www.rcsb.org.

[B49] Berman HM, Westbrook J, Feng Z, Gilliland G, Bhat TN, Weissig H, Shindyalov IN, Bourne PE (2000). The Protein Data Bank. Nucleic Acids Res.

[B50] Ceroni A, Passerini A, Vullo A, Frasconi P (2006). DISULFIND: a disulfide bonding state and cysteine connectivity prediction server. Nucleic Acids Res.

[B51] NetNGlyc 1.0 Server. http://www.cbs.dtu.dk/services/NetNGlyc.

[B52] Guex N, Peitsch M (1997). SWISS-MODEL and the Swiss-PdbViewer: An environment for comparative protein modeling. Electrophoresis.

